# Erosion of fish trophic position: an indirect effect of fishing on food webs elucidated by stable isotopes

**DOI:** 10.1098/rstb.2023.0167

**Published:** 2024-07-22

**Authors:** Davide Agnetta, Fabio Badalamenti, Christopher J. Sweeting, Giovanni D'Anna, Simone Libralato, Carlo Pipitone

**Affiliations:** ^1^ National Institute of Oceanography and Applied Geophysics (OGS), Trieste 34151, Italy; ^2^ NBFC, National Biodiversity Future Center, Palermo 90133, Italy; ^3^ CNR-IAS, Lungomare Cristoforo Colombo 4521, Palermo 90149, Italy; ^4^ School of Geosciences, University of Edinburgh, The King’s Buildings, Edinburgh EH9 3FE, UK; ^5^ Marine Management Organisation Lancaster House, Newcastle Business Park, Newcastle Upon Tyne NE4 7YH, UK; ^6^ CNR-IAS, Via Giovanni da Verrazzano 17, Castellammare 91014, Italy

**Keywords:** fishery-restricted areas, Mediterranean, stable isotopes, trawl ban, trophic position

## Abstract

Fishing has significant trophodynamic impacts on marine communities, including reductions in the mean trophic position (TP) of the ecosystem resulting from a decrease in the abundance and size of species and individuals with high TPs. This study demonstrates the erosion of fish TP, an additional process that results in lower TP of individuals of a given size, which may exacerbate the effects of fishing on the food web. A stable isotope approach based on the tRophicPosition Bayesian method was used to quantify the TP of 12 target marine species at a given length, and compare their TP between fishery-restricted areas and trawled areas. The results show a difference in the TP of six benthic and apical nekto-benthic predators, which feed in the median at about 0.5 TP lower in trawled areas. It appears that current ‘fishing down marine food webs’ analyses may underestimate the trophic effects of fishing. Accounting for changes in trophodynamics of individuals at a given size is important to detect indirect effects through food web interactions. The application of a trawling ban may lead to the restoration of lost trophic structure; however, trophic changes may occur more slowly than changes in biomass.

This article is part of the theme issue ‘Connected interactions: enriching food web research by spatial and social interactions’.

## 1. Introduction

Fisheries have consistently targeted large individuals and high trophic position (TP) species for human consumption resulting in their systematic depletion [1–3]. In some cases, the reduction of high TP species has led to an associated reduction in the mean TP of the entire marine community, a process referred to as the ‘fishing down the food web’ (FDFW) [4]. The FDFW has been questioned because of the use of fixed species-specific TPs [5], for being an apparent result of the serial addition of low TP species in fisheries targets [6], and for the non-ubiquitous declines in mean TP of fishery catches [7]. Nevertheless, there is increasing recognition of long-term trophodynamic changes in marine food webs induced by fisheries, especially those determined by biomass distribution across such fixed TP [3,8–11].

Fishing induces also disproportionate mortality on larger individuals, leading to a reduction in mean fish size within a population and thus in mean TP for a given species [12]. Therefore, not only the FDFW has been highlighted in several areas worldwide, but incorporating mean fish size reductions into analyses increases the rate at which trophic changes occur owing to fishing [13].

Fish size determines many aspects of predation behaviour, and larger individuals within a population commonly feed at a higher TP [14,15]. While incorporating size-specific feeding behaviour was a significant step forward, TP at a given size is still assumed to remain constant through time or in space, an assumption that is not necessarily true [16].

Spatial analyses of feeding behaviour emphasize significant plasticity within species that reflect the local availability of resources [17–19]. Such feeding plasticity has also been observed in freshwater and terrestrial systems where the TP of top predators increases with ecosystem size [20], where trophic roles change as a result of the introduction of new species [21] or of habitat fragmentation [18]. This suggests that for a given body size, feeding behaviour may vary based on the surrounding food resources and would be reflected in the TP of a consumer [22].

The exploitation of fishery resources has a wide range of impacts on marine ecosystems other than the reduction of mean fish body size, and this is particularly true for bottom trawling. These include changes in species richness [23,24], alteration of source production in supporting food webs [25,26], physical habitat degradation leading to structurally simpler bottom habitats [27,28], and impoverished benthic communities with subsequent reshuffle of trophic cascade processes [4,29,30].

Fishery-restricted areas (FRAs) can mitigate the direct and indirect impacts of fishery exploitation [31,32] and have proved useful as large field experiments for studying the ecosystem impacts of fishing on the abundance and size of organisms [29]. Moreover, FRAs have the potential for communities to recover from FDFW and to re-establish trophic interactions and TPs [33]. An example of FRAs in the Mediterranean is two trawl exclusion areas off the northern coast of Sicily, where many trawlable resources on the continental shelf have recovered significantly since the trawl ban [34,35].

**Figure 1.**
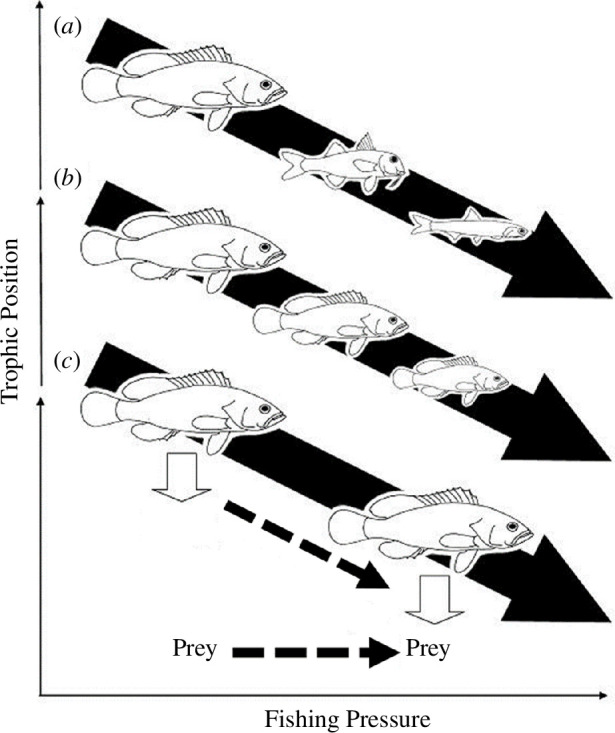
Representation of the three main mechanisms that lead to a reduction in the mean TP of marine food webs as some function of fishing pressure (i.e. fishing down marine food webs). (*a*) Change in species composition [4], (*b*) change in size structure of populations [13], and (*c*) change in TP of prey and/or prey composition for a given size of predator (this study: TP erosion).

Bottom-up and indirect effects of fishing on trophodynamics, such as those induced by changes in prey availability, have been shown in the entire food web of a central Mediterranean fishing area [36]. This suggests that another mechanism of TP reduction by fishing may occur, i.e. when the TP of predators of a given species and size is reduced indirectly as a result of changes in the traits of organisms lower in the food web (hereafter, TP erosion; figure 1). Nevertheless, few field studies have focused on such trophodynamic cascading changes at the species level of a given size, especially in large Mediterranean FRAs [33,37]. Understanding such changes is critical for predicting responses of food web components to exploitation and their potential recovery also for the vast use of the indicators based on TP [38].

Fish trophodynamics can be described using ^12^C/^13^C and ^14^N/^15^N stable isotope ratios. Such analyses rely on the relatively predictable enrichment in the heavy isotopes, which determines changes in isotopic ratio (δ) at each trophic step. Changes in δ^15^N are large and thus δ^15^N provides time-integrated estimates of TP. By contrast, changes in δ^13^C are relatively small but vary substantially among the different primary production sources that support food chains [39]. Moreover, using appropriate δ^13^C and δ^15^N values from food sources (baselines) is relevant for calculating the TP of specific consumers [16,40].

This study derives and compares isotope-based estimates of TP for demersal fish species at a given size caught in areas with different levels of fishing pressure. Assuming altered and unstable bottom conditions in trawled areas, we predict larger TP differences between FRAs and trawled areas in benthic fish feeders than in other feeding groups.

## 2. Material and methods

### (a) Sampling areas

**Figure 2.**
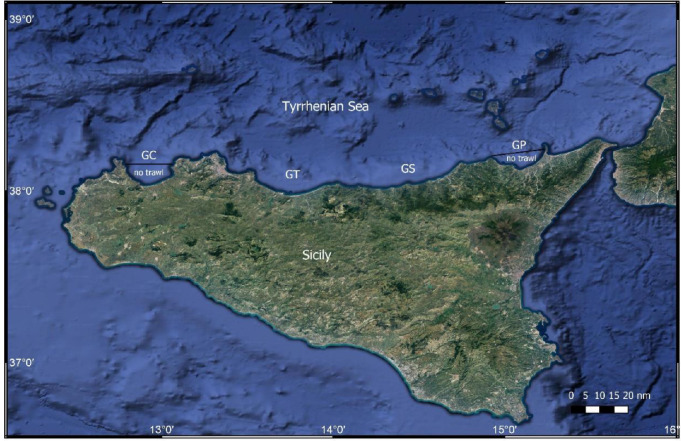
Map showing the four areas investigated: the no-trawl area in the Gulf of Castellammare (GC) and Patti (GP) and the two trawled areas of Gulf of Termini (GT) and Sant’Agata (GS).

Two FRAs and two trawled areas were surveyed in the southern Tyrrhenian Sea off the northern Sicily coast. The FRAs were the Gulfs of Castellammare (GC) and Patti (GP), while the trawled areas were the Gulfs of Termini Imerese (GT) and Sant’Agata (GS; figure 2). All four gulfs are characterized by the presence of river mouths, with a wide, gently sloping sandy bottom in the centre and by cliffs with vegetated rocky bottoms on the outer edges. Muddy bottoms dominate below 30 m depth. The four gulfs are subject to predominantly eastward currents following the general water mass circulation of the southern Tyrrhenian Sea. Satellite-derived annual mean chlorophyll *a*, sea surface temperature, and organic matter also show little variation among gulfs [41]. About 200 km^2^ of the continental shelf in GC and GP have been subject to a ban on commercial bottom trawling since 1990, allowing recovery of groundfish biomass and size and an average 8-fold biomass increase in GC [33,35,42]. Conversely, both GT and GS are exploited by large trawl fleets and are overfished [33,42]. In addition, they are characterized by lower diversity and lower biomass/abundance ratios of the macrobenthic assemblage compared to GC and GP [43].

### (b) Sampling of fishes and baseline for stable isotopes

Sampling was conducted in 2004–2005 and involved 12 fish species known as benthic, nekto-benthic and pelagic feeders, common to the four areas and targeted by fishermen (electronic supplementary material, table S1). These species represent about 40–50% of the total biomass in each gulf (unpublished data; Agnetta D., Pipitone C., 2005). Samples were taken from trawl-survey catches [44] or from local fishermen in the case of *Seriola dumerili* and *Dicentrarchus labrax*, stored in ice, and then returned to the laboratory within the same day. Total length was measured to collect similarly sized individuals at 50–60% of the maximum length as derived from Fishbase [45] (electronic supplementary material, figure S1).

Zooplankton and bottom-deposit feeding crustaceans are generally consumed by demersal fishes and have been used as relevant isotopic baselines in the previous studies conducted in the same areas [19,46,47]. Therefore, to assess the potential contribution of pelagic and benthic isotopic baselines to the TP estimates of fishes, mesozooplankton and suprabenthic crustaceans were collected in each gulf. Zooplankton (mainly copepods) were collected using a WP2 plankton net, while three benthic crustaceans (two decapods, *Alpheus glaber* and *Goneplax rhomboides* and one tanaid, *Apseudes spinosus*) were collected using a Van Veen grab. At least three individuals were collected for each fish species at a given size from each gulf, alongside three specimens for the baseline species. White dorsal muscle and the whole body were sampled for isotopic analysis from fishes and baseline invertebrates, respectively. Hydrochloric acid (HCl) was used to remove carbonates from baseline samples prior to isotope analysis. Because acid treatment may alter δ^15^N values [48], a half sample was washed with 2 N HCl and analysed for δ^13^C, the other half (untreated) was used to determine δ^15^N. All samples were rinsed with distilled water and oven dried at 60°C for approximately 48 h before being ground to a fine powder with a mortar and pestle. One-milligram samples of powdered tissue were then weighed into tin capsules for the determination of δ^13^C and δ^15^N using a PDZ Europa 20-20 elemental analyser with combustion and continuous flow isotope ratio mass spectrometer connected to an automated nitrogen–carbon analysis module for solid–liquid preparation (PDZ Europa Ltd, Northwich, UK). Two internal reference materials were analysed every six samples to compensate for instrument drift and to perform quality control. Isotopic signatures were expressed in conventional delta (δ) notation, referenced to the Atmospheric Air and PeeDee Belemnite international standards. Based on the s.d. of the internal standard, the precision of both δ^13^C and δ^15^N was ±0.2‰.

### (c) Bayesian trophic position calculation and baseline setting

TP of each of 12 fish species was calculated with a Bayesian model of the ‘tRophicPosition’ package [49] developed in R (v. 4.1.3 [50]). Briefly, the tRophicPosition package includes three different basic models depending on the number of baselines (i.e. benthic, pelagic, full) and the inclusion of a trophic discrimination factor (TDF) for carbon in addition to that for nitrogen. It works with stable isotope data (δ^13^C and δ^15^N values) for a robust estimation of consumer TP.

In order to develop the appropriate Bayesian model, we selected the relevant baseline and TDF [39]. The selection of baselines was performed in three steps. First, the variability of the isotopic signature (δ^13^C and δ^15^N) for pelagic and benthic taxa was compared using a two-way PERMANOVA based on the Bray–Curtis index to assess their use as a baseline. A symmetrical design with three factors: *trawl* with two levels (*yes*, *no*), *area* with two levels nested in *trawl* (GC, GP for level *no* and GS, GT for level *yes* of factor *trawl*) and *taxon* with four levels (meso-zooplankton and the three benthic species). The model obtained using 999 permutations and significance set at *p* = 0.05, showed a significant effect of the interaction *trawl* × *area* × *taxon* on δ^13^C and δ^15^N (*F*_6,81_ = 4.79, *p*<0.001), although the contribution to the total variance was greater for zooplankton than for the benthic organisms. Therefore, the analysis suggested to use separate species for each area (electronic supplementary material, figure S2). Second, we evaluated the inclusion of pelagic and benthic baselines in the model based on the distribution of fish isotopic signatures in relation to the four baselines using biplot graphs (electronic supplementary material, figure S2) as in [49]. Third, several tests were conducted to assess the best-fitting model (among the three alternatives), especially for more pelagic species (electronic supplementary material, figure S3). Based on the analysis of variance and biplots, a pelagic baseline (i.e. zooplankton) was not included in the model because (i) it was isotopically different from the other basal organisms so that it did not group together; (ii) the consumers never positioned between pelagic and benthic baselines. In fact, zooplankton was too depleted in δ^13^C (about 5‰) compared to the mean of fish isotopic signatures; and (iii) its contribution in the two-baseline models was not significant (electronic supplementary material, figure S3). Therefore, we calculated 5000 posterior Bayesian TPs (number of adaptation, burning and iteration 10^4^ with five chains) for each species in each gulf using the basic ‘one baseline’ model, as in equation (2.1), with the three benthic species from the same gulf as baseline:


(2.1) $$TP=\frac{\left(\delta^{15}N_{c}-\delta^{15}N_{b}\right)}{\Delta^{15}N}+\;\lambda .$$

TP is the trophic position of a consumer, δ^15^N_c_ and δ^15^N_b_ are the nitrogen isotopic values of the consumer and the baseline respectively and, λ is the TP of the baseline.

We used the common TDF proposed by Post [39] with a mean Δ^15^ *n* = 3.4 ± 0.98‰. The TP of the baseline, λ, is fixed and was set as default ( λ = 2), because we used primary consumers. It is worth noting that, since the model is additive with respect to λ, other choices for λ would affect absolute TP estimates for consumers but not their relative differences that are compared in this work. Based on the species-specific values reported by Fishbase for the selected species and preliminary model checks, prior distribution of fish TP was set to a mean of 4 with a s.d. of 0.5.

### (d) Trawling effect assessment on trophic positions

In order to test differences in TP for each species and for each pair of areas, the posterior TP Bayesian distributions (*n* = 5000 for each species and area) were used. To verify the difference in location parameters of the distribution (e.g. median) between areas with different trawling effort (i.e. FRAs versus trawled areas), a Kruskal–Wallis rank sum test was performed for each species. Shapiro–Wilk normality tests were carried out *a priori* for each dataset, and Wilcoxon pairwise tests (also called Mann–Whitney tests) were performed *a posteriori* for each combination of areas in pairs per species.

Moreover, quantile tests (‘snpar’ library) were performed to test for differences in the 95th percentile. Basic ranking of TP maxima for each species across area was used instead to assess the effects of trawling. All statistical analyses were performed using libraries available in the R environment [50].

## 3. Results

**Figure 3.**
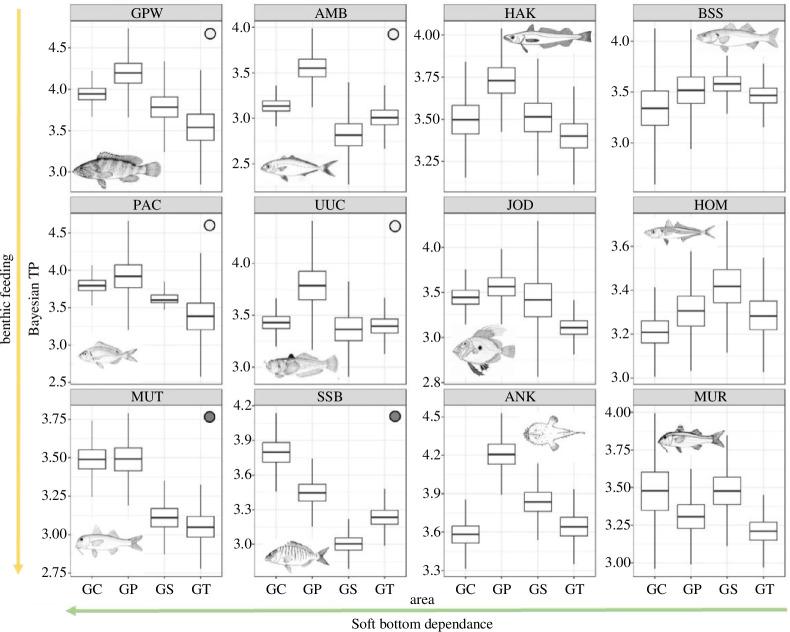
Box plots showing metrics (median, 25th and 75th percentile, minimum and maximum) of the posterior distribution of Bayesian TP (*n* = 5000) for the 12 species across areas. GC and GP are FRAs, GS and GT are trawl areas. Two directional axes were depicted to highlight the fish relationship with the macro-benthic prey (vertical axis) and their dependence on soft bottom (horizontal axis; see §§3 and 4 for details). Intermediate and strong responses of species to the trawl ban are indicated by light-grey and dark-grey dots, respectively (colours of dots refer to the significance of differences in TP, see details in table 1). GPW, white grouper; AMB, greater amberjack; HAK, hake; BSS, sea bass; PAC, pandora; UUC, stargazer; JOD, John Dory; HOM, horse mackerel; MUT, red mullet; SSB, sand steenbras; ANK, black-bellied anglerfish; MUR, striped red mullet.

Pairwise comparisons of TPs between FRAs (GC, GP) and trawled areas (GS, GT) showed significant differences in TP distributions for six species out of 12 (table 1; figure 3). Out of the species investigated, TPs of red mullet (MUT) and sand steenbras (SSB) were significantly higher in median, 95th percentile and maximum in FRAs compared to the trawled areas, although a significant difference resulted also between FRAs for SSB.

**Table 1.** Median and 95th percentile of posterior TP Bayesian distribution calculated for 12 species in two FRAs (GC, GP) and two trawl areas (GS, GT). (Pairwise tests were performed for all combinations of areas. *p*-values indicate significance of pairwise tests, ***p < 0.001, **p < 0.01, *p < 0.01. Species showing significant differences in median and 95th percentile between trawled areas and FRAs are coloured light grey, those also showing significant differences in TP maximum (see figure 3) in dark grey. Letters are in bold where pairwise tests showed both FRAs are significantly different from trawled areas.)

	median				Kruskall–Wallis and Wilcoxon			95th percentile				quantile test		
code	GC	GP	GS	GT	*χ* ^2^	pairwise	*p*	GC	GP	GS	GT	pairwise	*p*	exception
GPW	3.94	4.12	3.78	3.54	11 734.0	**GP**>**GC**>GS>GT	***	4.01	4.31	3.91	3.70	**GP**>**GC**>GS>GT	***	(*GC-GS)
AMB	3.14	3.55	2.82	3.01	13 911.0	**GP**>**GC**>GT>GS	***	3.19	3.65	2.94	3.09	**GP**>**GC**>GT>GS	***	
UUC	3.43	3.79	3.36	3.40	8233.2	**GP**>**GC**>GT>GS	***	3.49	3.92	3.48	3.46	**GP**>**GC**>GS>GT	***	(*GC-GS)
PAC	3.80	3.92	3.60	3.39	9525.7	**GP**>**GC**>GS>GT	***	3.86	4.07	3.67	3.56	**GP**>**GC**>GS>GT	***	
SSB	3.80	3.45	3.00	3.23	17 746.0	**GC**>**GP**>GT>GS	***	3.88	3.52	3.06	3.30	**GC**>**GP**>GT>GS	***	
MUT	3.49	3.49	3.11	3.05	15 024.0	**GC**=**GP**>GS>GT	***	3.55	3.56	3.17	3.12	**GP**>**GC**>GS>GT	***	(*GC-GS)
HAK	3.50	3.73	3.51	3.40	9935.2	GP>GS>GC>GT	***	3.58	3.80	3.60	3.47	GP>GS>GC>GT	***	(*GC-GS)
BSS	3.34	3.52	3.58	3.47	3466.9	GS>GP>GT>GC	***	3.51	3.65	3.65	3.54	GP>GS**>**GC>GT	***	(*GC-GS)
JOD	3.55	3.65	3.54	3.29	9806.4	**GP**>**GC**>GS>GT	***	3.62	3.73	3.68	3.38	GP>GS>GC>GT	***	(*GC-GS)
HOM	3.21	3.31	3.42	3.28	7484.7	GS>GP>GT>GC	***	3.26	3.37	3.49	3.35	GS>GP>GT>GC	***	(*GC-GS)
ANK	3.58	4.21	3.84	3.64	15463.0	GP>GS>GC>GT	***	3.65	4.29	3.91	3.71	GP>GS>GC>GT	***	
MUR	3.48	3.31	3.48	3.21	8879.6	GC=GS>GP>GT	***	3.60	3.39	3.57	3.27	GC>GS>GP>GT	***	(*GC-GS)

Greater amberjack (AMB), white grouper (GPW), pandora (PAC) and stargazer (UUC) had higher median and 95th percentile TP values in GC and GP; however, the maximum was not higher in each untrawled area.

TPs of sea bass (BSS) and John Dory (JOD) were lower in trawled areas but only in maximum and median values, respectively. All other species did not show a consistent pattern between trawled areas and FRAs in any of the metrics (table 1; figure 3).

Overall, the difference between trawled and untrawled areas was about 0.5 in median TP for five species and 0.2 for UUC.

## 4. Discussion

Differences in TP between trawled and FRAs for species at given size revealed indirect effects that could exacerbate fishing effects.

TP was approximately 0.5 higher in FRAs than in trawled areas, and differences were larger in selective benthic predators such as MUT, SSB, and PAC. At the size here investigated, these species scour the soft bottom in search of burrowing organisms, targeting their diet primarily on macrobenthic species such as crustaceans, polychaetes and bivalves [37,47].

Given the rapid response of macrobenthos to anthropogenic stressors such as bottom trawling [28], it is plausible that in disturbed areas, altered prey availability and organic matter depletion [43,51] indirectly led to changes in diet or feeding behaviour of benthic predators, which in turn were reflected in their lower TP.

The detritus and sediment in FRAs are probably different from those in areas open to trawling. In fact, mechanical disturbance and trawling in particular probably affect suspended particulate matter and seafloor biogeochemical processes [52], and in turn bacterial [53], macrozoobenthic [26,43] and fish [54] assemblages. In one of the investigated FRAs (i.e. GC), sediments are not subject to frequent resuspension and are probably richer in bacteria [51]. This would imply that food availability, as well as the production of primary food sources (i.e. algal versus bacterial) for benthic species, would differ between FRAs and trawled areas, perhaps contributing to the observed TP differences in predatory fishes. Hence, a process like niche compression with a reduction of the vertical dimension in isotopic space, occurring through multiple mechanisms as described for flowing agricultural streams [55], could also have occurred in the two Sicilian FRAs.

Higher TP in FRAs were also detected in nekto-benthic predators, such as GPW, greater AMB and UUC. The diet of these species is characterized by a high proportion of demersal fishes [33,56], the biomass of which has strongly increased in the FRAs investigated in this work [33,35]. This finding suggests a spread of the trawl effect to nekto-benthic feeders higher in the food web, mediated by a combination of changes in the availability of resources in the environment, which in turn have incorporated a TP change (in MUT for instance).

The TP of striped red mullet (MUR and of one of the main potential predators of MUT, black-bellied anglerfish (ANK) [33], did not show consistent differences in response to fishery impacts in the study areas (FRAs versus trawled areas). Although MUR is a benthic feeder anatomically very similar to MUT, it feeds on a mixture of habitats, including rocky substrates that are not hit (hence not importantly affected) by trawlers. The reason why ANK did not show any difference between untrawled and trawled areas is less clear. In fact, ANK probably fed on higher TP in GP compared to trawled areas, but not in GC. The species’ diet consists of decapod crustaceans and cephalopods in addition to fishes [57]; however, suprabenthic communities do not seem to be affected by trawling [41,58]. Authors [46] also found differences in the diets of suprabenthic-feeding juvenile scaldfish *Arnoglossus laterna* in three of our study areas (i.e. GC, GS and GT), but these differences could not be attributed to fishing pressure. In addition, few data are available on the diet of anglerfish but specimens in the 26–60 cm range (similar to the size studied here) are known to feed mainly on gadoids such as hake (HAK) [19,57,59], which showed no changes in TP in our gulfs. This supports our finding that there is no difference between trawled and untrawled areas for ANK.

A number of factors may explain why trawling did not impact the TP of species more involved in the pelagic food web. BSS, HAK, horse mackerel (HOM) and JOD feed also on pelagic fishes, which did not benefit from reduction in fishing mortality to the same extent as benthic prey [43], and on zooplankton that is less affected by trawling [58]. This could also depend on the capacity of larger-scale movements and a higher degree of omnivory by pelagic predators. Not only are these species less susceptible to the effects of trawling, but their isotopic signature may be the result of a mixed diet from different areas. By contrast, bottom trawl generally impacts benthic communities [28] and more sedentary species, making a TP change in the benthic food web more likely.

Our results suggest that significant trophodynamic changes have occurred in FRAs as a result of the trawl ban. Limited trophodynamic changes associated with fishery impacts were observed in MUT 9 years after the trawl ban [60]. The fact that similar or greater differences were observed for the same species in this study 15 years after the trawl ban suggests that it may take several years for trophic changes to occur as a consequence of reduced fishing impact. Analysis of gut contents also showed differences in adult *Ar. laterna* between GC and trawled areas [46]. Although trophodynamic effects of protection have not been observed in coastal Mediterranean marine protected areas (MPAs) [61,62], many factors such as their dimension and enforcement, the direct use of δ^15^N instead of the TP, and other confounding effects may have masked the expected result as already evidenced by the same authors.

We suggest that the TP erosion here observed in fish species at a given size may occur through a direct change in prey availability and size or through the incorporation of food web components (prey) that have in turn changed their TP as a result of bottom trawling. The same result could also potentially be induced by other mechanisms, such as when trophic fractionation differs among protection regimes. It was chosen as a widely accepted TDF for the investigated species and a change with other TDFs suggested for fishes could mainly determine variations in absolute TP values, while the analyses carried out here explore the relative differences.

This study assumes the same TDF in all areas. Lower quality food and ration size are associated with increased TDFs [63,64]; however, protein content and ration size are predicted to be higher in FRAs owing to higher availability of fish prey [33,42,65], thus, the changes in TP derived from the common TDF are in the opposed direction and potentially conservative for explaining the observed TP differences between FRAs and trawled areas in demersal species with a strong link to benthic prey.

TP erosion owing to trawl fishing, therefore, appears the most plausible process for explaining the observed patterns of TP changes for demersal species with strong benthic trophic relationships, although the mechanism by which the process occurs could not be identified from our data. Several additional mechanisms are likely in trawled areas including (i) reduced piscivory, (ii) the addition or loss of species lower in the food web, and (iii) changes in omnivory [55,66]. None of these are mutually exclusive and there is support for each. The relative magnitude of piscivory has been identified as one driver of TP change in relation to fishing [67]. Untrawled areas have been shown to have steeper abundance-size spectra and proportionally more abundant small fishes compared with trawled areas [42]. FRAs in general also exhibit greater species richness than fished areas and so the addition of new species to the food web is also a possible mechanism of extended food web length. Unfortunately, while stable isotopes can provide time-integrated estimates of diets that incorporate effects from variations in prey composition and size, they do not always represent a feasible solution to assess changes in the consumption of all prey species, especially from limited sample size. This could have detrimental effects in determining omnivory patterns of consumers, but a promising approach should come from the combination with DNA metabarcoding applied to gut contents [68].

Both stable isotope and gut content analyses are valuable tools in the assessment of marine trophodynamics, each with their own strengths and weaknesses. However, it is unlikely that TP erosion would be easily detected with gut content analyses only, mainly because the effort required to obtain gut content data across multiple locations, several species and their prey would be huge. The value of gut content analysis however, would be in refining the understanding of the mechanisms driving the TDF, testing for example increased piscivory or changes in prey size distribution. A further positive contribution in revealing TP erosion could come from compound-specific isotopic analysis, which overcomes issues related to limited sample size and selection of baselines [69].

Our findings have a number of implications for management that highlight the potential of MPAs, such as FRAs for fisheries management and ecosystem rehabilitation, and suggest that successful MPAs may be built on the elimination of fishing gear that impact benthic habitats rather than just a blanket no-take approach. Such a fisheries management tool has wide potential, especially in subtropical areas like the Mediterranean, where fishing bans like those generally implemented in MPAs are deemed a highly promising management approach in multispecies fisheries [35,70] and for a practical assessment of the status of marine ecosystems as required by regulations such as the Marine Strategy Framework Directive [38].

Although our findings have to be confirmed by studies at larger scale, we provide evidence of an additional process by which fishing may indirectly impact trophic structure and functional diversity and suggest that fishing effects may be more severe and complex than previously thought.

## Data Availability

The datasets supporting this article are within the paper or have been uploaded as part of the electronic supplementary material [71].
